# Disseminated Nocardia Farcinica Pneumonia with Left Adrenal Gland Abscess

**DOI:** 10.7759/cureus.1160

**Published:** 2017-04-12

**Authors:** Christopher Jackson, Brennan McCullar, Kiran Joglekar, Ankur Seth, Hiren Pokharna

**Affiliations:** 1 Internal Medicine, University of Tennessee Health Science Center Memphis; 2 Medicine, University of Tennessee Health Science Center Memphis

**Keywords:** adrenal gland, pulmonary nocardiosis, fever of unknown origin, pneumonia

## Abstract

Adrenal masses pose a diagnostic challenge. The differential diagnosis includes functional adrenal tumors, incidentally found adrenal masses, metastases from an unknown primary cancer, and abscesses. Infrequently, adrenal gland abscesses have been reported in disseminated nocardiosis affecting immunocompetent and immunocompromised patients. We report a case of disseminated Nocardia farcinica pneumonia with an adrenal gland abscess in an immunocompetent patient with no identified risk factors for nocardiosis.

## Introduction

Adrenal masses are found in less than 1% of imaging studies done for indications other than primary imaging of the adrenal glands [1]. Most adrenal masses are non-functional adenomas, although hormonally-secreting tumors, metastases, and abscesses are additional considerations [2]. Rarely, adrenal masses can come from infectious causes, especially if bilateral abscesses are present [3]. Disseminated nocardiosis is an uncommon cause of adrenal gland abscesses. We report a case of unilateral left adrenal gland abscess from disseminated Nocardia farcinica pneumonia in an immunocompetent host. 

## Case presentation

A 69-year-old Caucasian male with a history of coronary artery disease and tobacco dependence initially presented to his primary care physician with fevers up to 103° Fahrenheit and nocturnal hyperhidrosis for six weeks. Associated symptoms included intermittent chest pain, facial flushing, paroxysmal hypertension, involuntary weight loss, left upper abdominal pain, and diarrhea. The patient was a retired pilot with a recent history of volunteering at prisons. He also traveled to Israel eight months prior to presentation. His physical examination was unremarkable. Routine laboratory studies were only significant for leukocytosis. Chest x-ray showed minimal interstitial changes. He was started on antibiotic therapy with azithromycin for possible atypical pneumonia. His symptoms improved for four to five days before returning.

**Figure 1:**
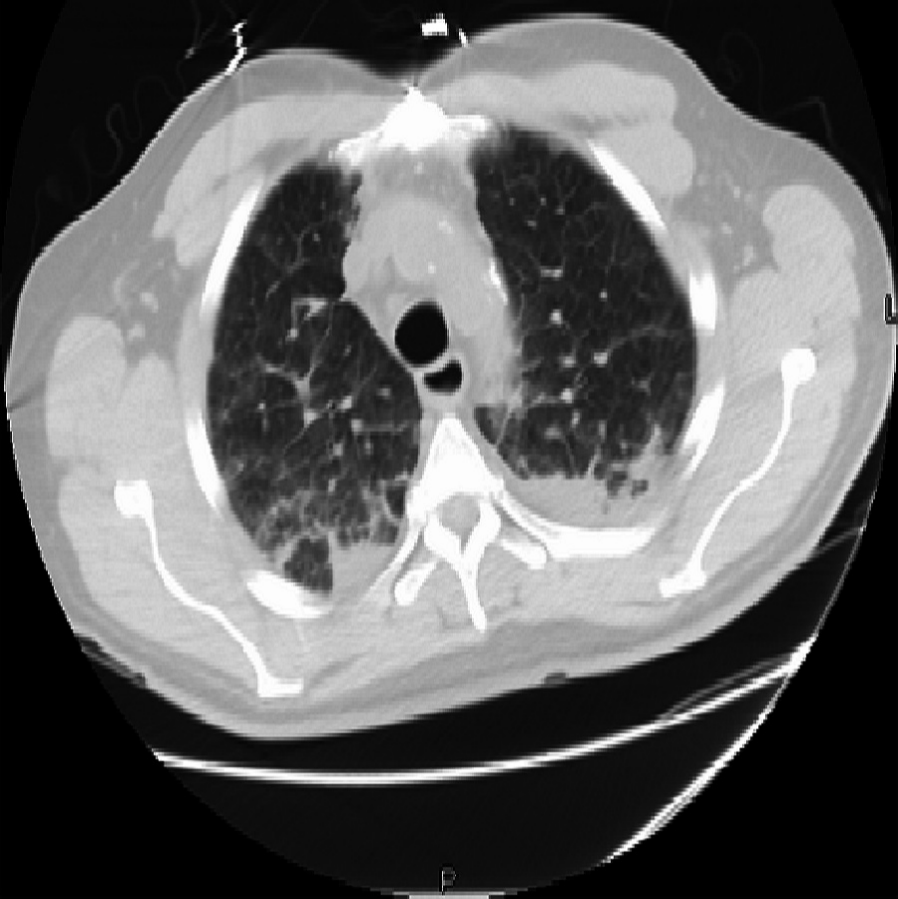
Computed tomography of the chest Ground glass changes in the mid-lung zones bilaterally, along with bilateral pleural effusions

**Figure 2:**
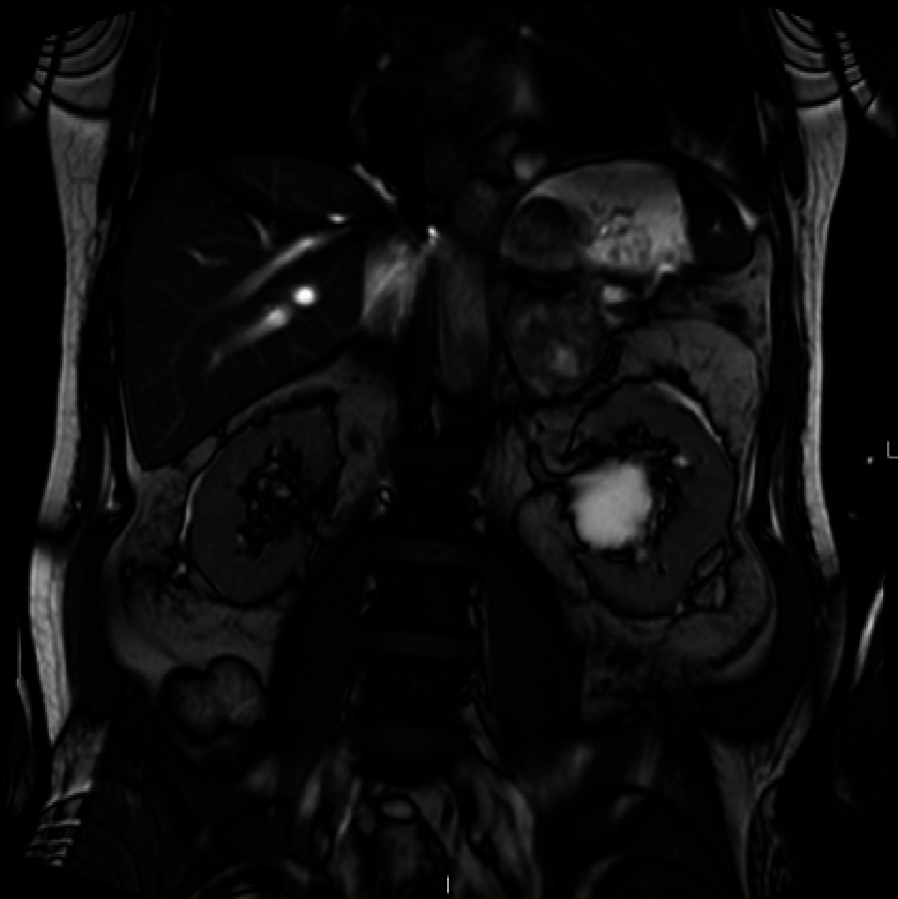
Computed tomography of the abdomen and pelvis Left heterogenous adrenal gland mass concerning for hemorrhage

Given the persistent fever and leukocytosis (white blood cell count of 15,200 cells per microliter with 88.5% neutrophils), he underwent further testing for human immunodeficiency virus (HIV), tuberculosis, and possible occult malignancy. His purified protein derivative, interferon gamma release assay, and HIV tests were all negative. Computed tomography (CT) of the chest showed minimal interstitial changes and bilateral pleural effusions (Figure 1). A CT of the abdomen and pelvis showed a 4 cm irregular left adrenal mass (Figure 2). Given the new adrenal mass and the patient’s symptoms, he was admitted to the hospital for further evaluation.

Patient’s admission vital signs and physical examination were within normal limits. Laboratory studies were significant for leukocytosis at 28,600 white blood cells per microliter with a left shift of 93.9% neutrophils. Multiple blood and urine culture samples were obtained, and the patient was initially observed off of antibiotics. Rheumatologic and fungal serologies, including an antinuclear antibody, anti-cytoplasmic nuclear antibody, rheumatoid factor, citric citrullinated peptide, and 1-3 beta-d-glucan, were negative. Functional studies obtained, including blood and urine catecholamines and metanephrines, were unremarkable. The patient’s cortisol level failed to suppress with a low-dose dexamethasone test. However, further workup with a high-dose dexamethasone suppression test showed no evidence of Cushing's disease. Given his worsening respiratory status after admission, he was placed on broad-spectrum antimicrobial coverage with piperacillin-tazobactam - 4.5 mg intravenous (IV) every eight hours, linezolid - 600 mg IV every 12 hours, amphotericin B - 5 mg per kg IV daily, and azithromycin - 500 mg IV every 24 hours.

For further evaluation of malignancy, he underwent a bone-marrow biopsy that was negative for neoplastic cells or evidence of granuloma formation. After the bone marrow biopsy, the patient developed acute respiratory failure requiring facemask ventilation. Repeat CT of the chest showed bilateral basilar consolidation and pulmonary edema suggestive of multifocal pneumonia. His blood, urine, and bone marrow cultures returned positive with Gram-positive rods, and these cultures were sent for modified acid-fast stain. He was started on IV trimethoprim-sulfamethoxazole (TMP-SMX), 15 mg/kg every six hours, and meropenem, 500 mg every six hours, given concern for nocardiosis, and he defervesced with significant clinical improvement. A transthoracic echocardiogram revealed no vegetation or mass.

He continued to improve prior to discharge. However, he then developed recurrent left upper quadrant abdominal pain. Repeat CT of the abdomen and pelvis showed a left adrenal mass with irregular peripheral enhancement, celiac axis soft tissue extension, and thrombus extending from the left adrenal vein into the left renal vein, all highly suspicious for malignancy. As all the testing done essentially ruled out pheochromocytoma, the adrenal mass was biopsied. The adrenal biopsy returned positive for Nocardia farcinica species. His final blood, urine, and bone marrow cultures also resulted as Nocardia farcinica. The patient was discharged on parenteral meropenem, 1 gm every eight hours, and oral TMP-SMX 800/160 mg, two tablets twice a day for eight weeks. After eight weeks, the patient would continue oral TMP-SMX 800/160 mg, two tablets twice a day for nine months. Follow-up imaging would be based on clinical progress. 

At outpatient follow-up, magnetic resonance imaging (MRI) of the brain was performed showing two brain lesions; the findings were not surprising, given the proclivity of disseminated nocardiosis to involve the brain. The patient was re-admitted for further workup of his new brain lesions. A transesophageal echocardiogram performed did not show evidence of vegetation or mass. Neurosurgery evaluated the patient and recommended continued medical management, given there were no neurologic deficits. He is continuing intravenous meropenem and oral TMP-SMX with plans to transition to chronic oral TMP-SMX therapy as guided by the infectious disease specialists.

## Discussion

Nocardia are ubiquitous Gram-positive, rod-shaped, filamentous, partially acid-fast organisms found in soil. Over 80 species of Nocardia have been identified with 33 being pathogenic in humans [4]. The clinical spectrum of disease includes self-limiting subclinical pneumonia to life-threatening disseminated infection [4]. The infection starts in the lungs with an acute or subacute resolving pneumonitis. Patients with symptomatic pulmonary Nocardia infection are frequently immunosuppressed [5].

Disseminated nocardiosis occurs more often in immunocompromised than immunocompetent hosts [5]. The most common site for metastasis is the central nervous system (CNS). However, the metastatic spread can occur to any organ [6]. Adrenal gland involvement with the Nocardia infection is rare with seven cases reported in the English literature upon performing a multiple database literature review using the terms "disseminated nocardiosis" and "adrenal gland" [4-10]. The rarity of cases reflects the challenge of determining the etiology of adrenal masses and the slow growth of Nocardia in a culture medium.

Characteristics of previous cases of adrenal gland abscess in disseminated nocardiosis are outlined in Table 1. Median age at the time of diagnosis was 49 years. Females were more commonly affected than males. Females were also more likely to die from disseminated infection compared to males. Paradoxically, infection with Nocardia farcinica occurs three times more often in males compared to females. Nevertheless, few deaths have been reported due to Nocardia adrenal gland abscesses [4-10].

**Table 1:** Prior Cases of Adrenal Abscesses Associated with Disseminated Nocardiosis AIDS - acquired immune deficiency syndrome; RA - rheumatoid arthritis; TNF - tumor necrosis factor; TPN - total parenteral nutrition; HD - hemodialysis; IVC - inferior vena cava; CNS - central nervous system

No.	Reference	Age/Sex	Presenting symptoms	Predisposing Factor	Abscess Location	Metastatic spread	Adrenal intervention?	Nocardia spp.	Death
1	Kim, et al. [9]	38/Male	Left upper quadrant abdominal pain, fever, chills	Yes (AIDS)	Left	Paraaortic and mesenteric lymphadenopathy	Laparoscopic drainage	Asteroides	Yes
2	Arabi, et al. [7]	39/Male	Epigastric pain, fever, vomiting	Yes (AIDS)	Bilateral	CNS	None	Asteroides	No
3	Midiri, et al. [10]	49/Female	Fever, left flank pain	Yes (chronic steroids for RA)	Left	Spleen	Adrenalectomy	Asteroides	No
4	Chong, et al. [8]	34/Male	Fever, left loin pain, hematuria	Yes (AIDS)	Left	None	Laparoscopic drainage	Asteroides	No
5	Tachezy, et al. [5]	71/Female	Fever, non-productive cough, adynamia	Yes (malnutrition)	Right	Right hepatic capsule, right renal capsule, IVC, CNS	Adrenalectomy with resection of IVC, diaphragm, retroperitoneum, and Gerota’s fascia	Farcinica	No
6	Al Tawfiq, et al. [4]	66/Female	Fever, left upper quadrant pain	Yes (TNF -alpha therapy for chronic psoriasis)	Bilateral	Left renal vein, spleen, retroperitoneal & mesenteric lymphadenopathy	None	Farcinica	Yes
7	de Montmollin, et al. [6]	59/Female	Fever, right lumbar region pain	Yes (chronic TPN, HD, malnutrition)	Right	None	Laparoscopic drainage	Farcinica, araoensis, otitidiscaviarum	Yes
8	This case	69/Male	Fever, night sweats, left upper quadrant abdominal pain	No	Left	CNS, bone marrow, left renal vein	None	Farcinica	No

Most patients presented with fever and/or abdominal pain on the side of adrenal gland involvement. All but one of the previous cases had a predisposing factor. Immunosuppression and malnutrition are the most well-described risk factors for disseminated nocardiosis [6]. Metastatic spread occurred in five out of seven cases, ranging from the ipsilateral renal vein to the CNS. Most patients had progression of infection despite appropriate antimicrobial therapy. Only one patient had adrenalectomy while others had laparoscopic drainage or antibiotics only. There are no consensus guidelines on doing adrenalectomy vs. laparoscopic drainage for disseminated nocardiosis involving the adrenal gland.

Review of prior cases shows that the Nocardia asteroids complex causes most cases of nocardiosis worldwide [6]. Nocardia farcinica appears to be the most virulent member of this group, causing more disseminated disease [4]. With nocardiosis caused by the farcinica species, mortality is 35% with previously published cases. Mortality can approach 55% with cerebral involvement [4]. Treatment of choice is TMP-SMX if Nocardia spp. is susceptible [5]. The overall prognosis for disseminated nocardiosis is good if the patient receives appropriate therapy early.  

## Conclusions

Our case highlights a few important clinical pearls. Nocardiosis should be suspected in patients with possible subclinical pneumonia and abnormal adrenal masses present. This consideration is independent of the immune status of the patient. When nocardiosis is suspected, empiric treatment with parenteral TMP-SMX is indicated, given the increased mortality risk. Prolonged antibiotic therapy with TMP-SMX is necessary to achieve eradication of Nocardia. Patients who receive treatment early can have a good prognosis, as in our case. 
